# Ultrasonic Vocalizations Emitted during Defensive Behavior Alter the Influence of the Respiratory Rhythm on Brain Oscillatory Dynamics in the Fear Circuit of Rats

**DOI:** 10.1523/ENEURO.0280-19.2019

**Published:** 2019-08-22

**Authors:** Rosalind S.E. Carney

## Abstract

**Highlighted Research Paper:** New Insights from 22-kHz Ultrasonic Vocalizations to Characterize Fear Responses: Relationship with Respiration and Brain Oscillatory Dynamics, by Maryne Dupin, Samuel Garcia, Julie Boulanger-Bertolus, Nathalie Buonviso, and Anne-Marie Mouly

Vocalization by animals enables intraspecies communication about dynamic environmental conditions, such as the presence of predators, food availability or scarcity, and also facilitates nurturing interactions from mother to offspring. Vocalizations conform to the auditory range of each species and can vary with age (Portfors, 2007). For example, rat pups emit 40-kHz ultrasonic vocalizations (USVs) when they are separated from their mothers, and these oscillations have been used as an indicator of distress in studies examining the effects of maternal neglect (Simola, 2015; Courtiol et al., 2018). Adult rats emit either 22-kHz or 50-kHz USVs, which reflect negative and positive emotional states, respectively (Portfors, 2007). Rats emit 22-kHz USVs when they are exposed to aversive stimuli, such as foot shocks (Wöhr et al., 2005), which serve as an unconditioned stimulus in fear conditioning experiments. Inaudible to humans, 22-kHz USVs can be recorded and used as an indicator of emotional status in combination with overt fear responses such as escape or freezing (absence of body movement excluding respiration) behaviors. As opposed to escape, freezing is a passive response and is frequently used as an indicator of fear during animal experimentation.

Freezing behavior alters brain oscillations; the onset and offset of freezing is temporally related to sustained 4-Hz oscillations in the medial prefrontal cortex–basolateral amygdala (mPFC–BLA) circuitry (Dejean et al., 2016; Karalis et al., 2016). These oscillations are distinct from the theta (4–12 Hz) and gamma (30–120 Hz) synchronized oscillations in mPFC–BLA circuitry that occur during the memory consolidation and retrieval components of fear expression (Seidenbecher et al., 2003; Popa et al., 2010; Headley and Paré, 2013; Herry and Johansen, 2014; Likhtik et al., 2014; Stujenske et al., 2014; Bocchio et al., 2017). Synchronized oscillations are thought to facilitate coordination of activities between neuronal populations and can be influenced by an animal’s respiratory rate (Heck et al., 2017; Tort et al., 2018). As 22-kHz USVs are produced during exhalation and decrease the respiratory rate (Frysztak and Neafsey, 1991; Hegoburu et al., 2011; Sirotin et al., 2014; Boulanger-Bertolus et al., 2017), they can also affect oscillatory patterns in the brain. An understanding of the interplay of USV production, freezing behavior, and respiration on brain oscillatory dynamics—which could have an impact on the interpretation of fear expression parameters—was lacking. In their *eNeuro* publication, Dupin et al. (2019) investigated how USV production by a rat alters brain oscillations in its fear expression circuit and how these changes relate to the rat’s respiratory cycle.

The experimental approach required simultaneous recording of changes in respiration and brain oscillations and recording of 22-kHz USVs in adult rats. This was facilitated by the use of a customized whole-body plethysmograph, an instrument used to record pressure changes due to an animal’s respiration, which was described in a previous study (Hegoburu et al., 2011). In addition to measuring respiration, the customized chamber contains a microphone and video cameras to facilitate recording USV production and body movements, respectively. To enable a fear conditioning paradigm, in which rats learn to associate an odor with a foot shock, the chamber also has an electrified floor and tubing in the ceiling of the chamber through which odors can be dispensed.

Adult rats underwent a surgical procedure to implant local field potential (LFP) wireless recording electrodes in three locations in the left hemisphere of the brain: BLA, mPFC, and the piriform olfactory cortex (PIR); the PIR is involved in odor fear conditioning. A 2 week recovery period was followed by 4 d of brief (30 min) acclimatization to the customized chamber. During fear conditioning, each rat was placed in the chamber for 4 min before the onset of the first of 10 odor-shock trials that were separated by 4 min intertrial intervals. In each trial, an odor was dispensed into the cage for 20 s, and a foot shock was delivered during the last second of each trial. In the minute after the foot shock, the rats exhibited either freezing or escape behavior. As expected, rats emitted USVs while exhibiting freezing behavior. An unexpected finding was that USVs were also emitted during escape behavior, albeit to a lesser extent than during freezing behavior. This led the authors to further define the 1-min period of post-foot shock behavior into four categories: USV freezing, silent freezing, USV escape, and silent escape. USVs emitted during escape were louder and of shorter duration than the USVs emitted during freezing.

Fear retention was tested 48 h after fear conditioning. Each rat was placed in the customized chamber that now contained new visual cues and a plastic floor to eliminate contextual fear. After 4 min of acclimatization, the odor was presented for 20 s, for a total of five times, separated by 4 min intertrial intervals. Defensive behavior during the 20 s odor presentation period was measured and averaged over the trials. This analysis revealed that the amount of USV freezing during fear conditioning was positively correlated with the amount of USV freezing during the fear retention test. No such relationship was found for escape behavior. The observations thus far showed that rats emit USVs of different duration and amplitude during passive or active defensive behaviors. Only USVs emitted during freezing were predictive of the fear response during retention testing when the original unconditioned stimulus was no longer present.

Brain oscillatory dynamics in the BLA, mPFC, and PIR during the 1-min post-foot shock period during fear conditioning were temporally related to periods of USV calls; individual USVs were of too short a duration to permit individual USVs to be directly linked to neural activity. Power spectral density (PSD) is the time-frequency representation in which mean power signals are separated into delta (0–5 Hz), theta (5–15 Hz), beta (15–40 Hz), and gamma (40–80 Hz) frequencies. Compared with silent periods in which no USVs were emitted, periods of USV vocalization, during either freezing or escape behavior, coincided in increases in delta, beta (in PIR), and gamma oscillations, and a decrease in theta oscillations in all recorded regions (Fig. 1). These results show that USV production affects oscillatory dynamics in the fear expression circuit.

**Figure 1.**
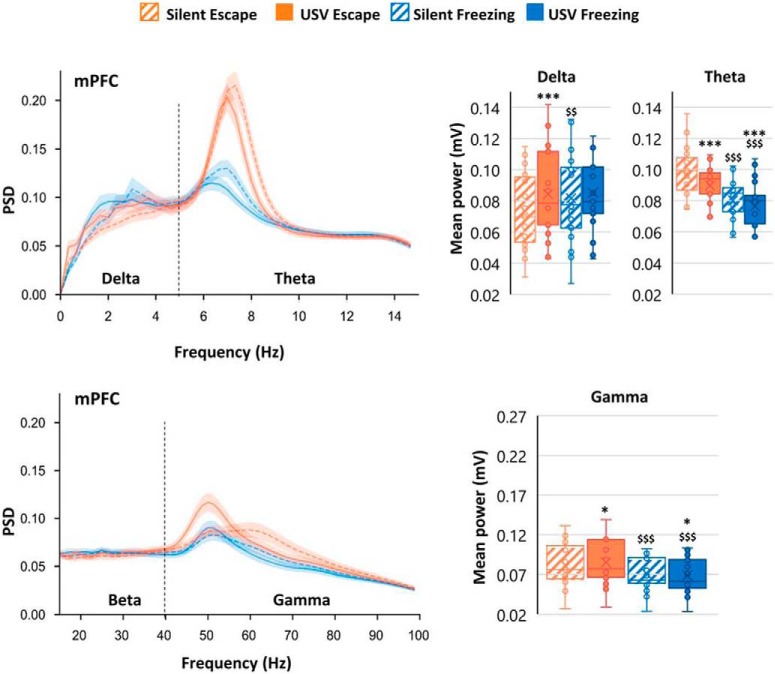
PSD of mPFC LFP signals and mean power in delta (0–5 Hz), theta (5–15 Hz), and gamma (40–80 Hz) bands. The average PSD (±SEM) is represented on the left, and the delta, theta, and gamma average power (±SEM) is represented on the right. *p < 5 × 10^−2^, **p < 5 × 10^−3^, ***p < 5 × 10^−4^: significant difference between same color-different pattern bars; $*p* < 5 × 10^−2^, $$*p* < 5 × 10^−3^, $$$*p* < 5 × 10^−4^: significant difference between same pattern-different color bars. (Adapted from Figure 3 & Figure 4 in Dupin et al., 2019).

Brain oscillatory changes were next related to changes in respiration parameters. First, USV emission uncoupled the relationship, which was present during silent freezing, between delta frequency in the recorded brain regions and the respiratory frequency (Fig. 2). Second, USV emission induced a reorganization of beta and gamma activity power during the respiratory cycle, with increased beta power during the first half of expiration phase, and increased gamma power during the second half of expiration (Fig. 3). These results show that some of the effects of USV emission on brain oscillatory dynamics are mediated by its modulation of respiratory signal.

**Figure 2.**
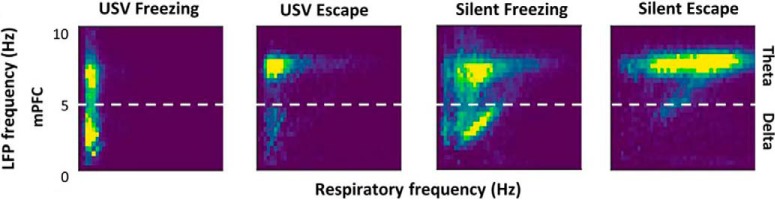
Covariation between delta and theta oscillatory frequencies and respiratory frequency. 2D matrix histograms obtained from LFP signals recorded in mPFC; *y*-axis represents LFP frequency and *x*-axis represents respiratory frequency. The 2D histogram is normalized so that the total sum is 1, and point density is represented on a color scale ranging from blue to yellow as the point density increases. (Adapted from Figure 6 in Dupin et al., 2019).

**Figure 3.**
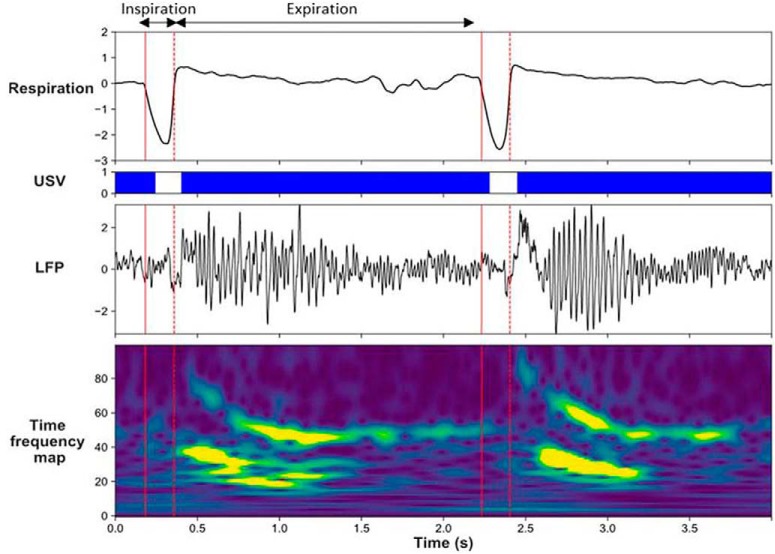
Modulation of beta and gamma power by the phase of the respiratory cycle. Individual traces representing from the top, respiratory signal, USV calls, raw LFP signal recorded in the PIR and its time frequency map (*y*-axis, LFP signal frequency in Hz; *x*-axis, time in milliseconds). (Adapted from visual abstract in Dupin et al., 2019).

This *eNeuro* publication by Dupin et al. (2019) includes several findings applicable to various disciplines of neuroscience. The authors show that USVs are emitted during escape behavior. Whereas USV production during locomotion had been previously described (Laplagne and Elías Costa, 2016; Boulanger-Bertolus et al., 2017), the results of the current study suggest that USV production cannot be wholly associated with the passive defensive behavior of freezing—USV production occurs to a small extent during the active defensive behavior of escape. The authors also show that USV emission and defensive behavior can modulate the various oscillatory dynamics of the brain fear circuit, in part, by influencing the respiratory cycle. These observations suggest that different manners of fear expression can contribute to respiration-induced alterations in brain oscillations, and in turn, cognitive function. The authors hypothesize that USV-induced alterations of synchronous neural activity in fear circuitry could reinforce the strength of fear memories. Future studies in Dr. Mouly’s laboratory (Lyon Neuroscience Research Center, INSERM U1028, Lyon, France) will investigate whether fear memory is altered when certain brain structures involved in USV production are inactivated. A better understanding of the impact of USV production on brain neural dynamics is particularly relevant for rodent models of human neurodevelopmental disorders for which socio-affective communication is severely impaired, such as autism spectrum disorder (Wöhr and Scattoni, 2013).
